# Association of *MTHFR* C677T Genotype With Ischemic Stroke Is Confined to Cerebral Small Vessel Disease Subtype

**DOI:** 10.1161/STROKEAHA.115.011545

**Published:** 2016-02-22

**Authors:** Loes C.A. Rutten-Jacobs, Matthew Traylor, Poneh Adib-Samii, Vincent Thijs, Cathie Sudlow, Peter M. Rothwell, Giorgio Boncoraglio, Martin Dichgans, James Meschia, Jane Maguire, Christopher Levi, Natalia S. Rost, Jonathan Rosand, Ahamad Hassan, Steve Bevan, Hugh S. Markus

**Affiliations:** From the Department of Clinical Neurosciences, University of Cambridge, Cambridge, UK (L.C.A.R.-J., M.T., H.S.M.); Department of Medical & Molecular Genetics, King’s College London, London, UK (M.T.); Stroke and Dementia Research Centre, Department of Clinical Neuroscience, St George’s University of London, London, UK (P.A.-S.); KULeuven Department of Experimental Neurology and Leuven Research Institute for Neuroscience and Disease, University of Leuven, and Laboratory of Neurobiology, Vesalius Research Center, VIB, Leuven, Belgium and Department of Neurology, Austin Health and Florey Institute of Neuroscience and Mental Health, Heidelberg, Australia (V.T.); Division of Clinical Neurosciences, Neuroimaging Sciences and Institute of Genetics and Molecular Medicine, University of Edinburgh, Edinburgh, UK (C.S.); Stroke Prevention Research Unit, Nuffield Department of Neuroscience, University of Oxford, UK (P.M.R.); Department of Cerebrovascular Diseases, Fondazione IRCCS Istituto Neurologico “Carlo Besta”, Milano, Italy (G.B.); Institute for Stroke and Dementia Research, Klinikum der Universität München, Ludwig-Maximilians-University Munich and Munich Cluster of Systems Neurology, SyNergy, Munich, Germany (M.D.); Department of Neurology, Mayo Clinic, Jacksonville (J.M.); School of Nursing and Midwifery (J.M.) and Hunter Medical Research Institute (J.M., C.L.), University of Newcastle, NSW, Australia; Center for Human Genetic Research and Department of Neurology, Massachusetts General Hospital, Boston (N.S.R., J.R.); Department of Neurology, Leeds General Infirmary, Leeds Teaching Hospitals NHS Trust, Leeds, UK (A.H.); and School of Life Science, University of Lincoln, Lincoln, UK (S.B.).

**Keywords:** cerebral small vessel disease, genetic association, homocysteine, hypertension, lacunar stroke, MTHFR

## Abstract

Elevated plasma homocysteine levels are associated with stroke. However, this might be a reflection of bias or confounding because trials have failed to demonstrate an effect from homocysteine lowering in stroke patients, although a possible benefit has been suggested in lacunar stroke. Genetic studies could potentially overcome these issues because genetic variants are inherited randomly and are fixed at conception. Therefore, we tested the homocysteine levels–associated genetic variant *MTHFR* C677T for association with magnetic resonance imaging–confirmed lacunar stroke and compared this with associations with large artery and cardioembolic stroke subtypes.

**Methods—:**

We included 1359 magnetic resonance imaging–confirmed lacunar stroke cases, 1824 large artery stroke cases, 1970 cardioembolic stroke cases, and 14 448 controls, all of European ancestry. Furthermore, we studied 3670 ischemic stroke patients in whom white matter hyperintensities volume was measured. We tested *MTHFR* C677T for association with stroke subtypes and white matter hyperintensities volume. Because of the established association of homocysteine with hypertension, we additionally stratified for hypertension status.

**Results—:**

*MTHFR* C677T was associated with lacunar stroke (*P*=0.0003) and white matter hyperintensity volume (*P*=0.04), but not with the other stroke subtypes. Stratifying the lacunar stroke cases for hypertension status confirmed this association in hypertensive individuals (*P*=0.0002), but not in normotensive individuals (*P*=0.30).

**Conclusions—:**

*MTHFR* C677T was associated with magnetic resonance imaging–confirmed lacunar stroke, but not large artery or cardioembolic stroke. The association may act through increased susceptibility to, or interaction with, high blood pressure. This heterogeneity of association might explain the lack of effect of lowering homocysteine in secondary prevention trials which included all strokes.

## Abstract 2

Supplemental Digital Content is available in the text.

Elevated plasma homocysteine levels (tHcy) have been consistently associated with the risk of ischemic stroke in observational studies.^1^ Moreover, experimental studies suggest that increases in total homocysteine aggravates vascular disease.^2^ However, some clinical trials that investigated the benefit of lowering tHcy with B vitamins to reduce the risk of stroke have been negative.^3–6^ In contrast, a recent large primary prevention trial in China, the China Stroke Primary Prevention Trial (CSPPT), which recruited only hypertensive patients, demonstrated a beneficial effect in reducing risk of stroke.^7^ Possible reasons for this conflicting evidence include insufficient stroke phenotyping particularly if homocysteine is a predominant risk factor for one type of stroke, dietary folate fortification reducing tHcy in populations in which trials have been performed, interactions between treatment and risk factors, and insufficient treatment duration.

A subtype-specific effect, with elevated homocysteine primarily increasing risk for small vessel disease (SVD) stroke, has been suggested by both epidemiological data and secondary analysis of clinical trials. Case–control studies have suggested that elevated homocysteine is primarily a risk factor for lacunar stroke^8,9^ and that there may be heterogeneity even within this subtype, with strongest associations in those SVD cases with multiple lacunar infarcts and confluent leukoaraiosis on magnetic resonance imaging (MRI).^10^ Most previous clinical trials that investigated the benefit of lowering homocysteine studied stroke as a combined event lumping together all different etiologies (ie, hemorrhage and ischemic and ischemic stroke subtypes). A secondary analysis in the Vitamins to Prevent Stroke (VITATOPS) trial^3^ found a borderline treatment effect in patients with lacunar stroke (hazard ratio 0.80 (95% confidence interval [CI] 0.67–0.96)), whereas an MRI VITATOPS substudy found vitamin-lowering therapy was associated with reduced white matter lesion volume progression in patients with severe white matter lesions.^11^ A further possibility is that elevated homocysteine may interact with certain cardiovascular risk factors, and treatment effect may only be detected if these interactions are taken into account; of possible relevance, the positive CSPPT trial was only performed in hypertensive individuals.

Another possible explanation for the conflicting epidemiological and clinical trial data is that the association between homocysteine and risk of ischemic stroke is a reflection of reverse causality or residual confounding, that is, elevated tHcy does not play a causal role in stroke pathogenesis but are merely noncausally associated with an increased risk of stroke. Genetic studies have the potential to overcome these issues by using genetic variants associated with elevated tHcy as a proxy for tHcy because the inheritance of genetic variants is random and not influenced by confounding factors. The most often studied genetic variant, showing the strongest association with increased tHcy, is the cytosine (C) to thymine (T) substitution at position 677 of the methylene tetrahydrofolate reductase (*MTHFR*) gene (rs1801133).^12–14^ Case–control studies that investigated the association of the *MTHFR* C677T variant with stroke yielded inconsistent results, which is likely because of small sample sizes and the varying stroke phenotypes studied. Meta-analyses have produced conflicting results with an association reported between *MTHFR* and ischemic heart disease and stroke in one study but not with stroke in another.^12,15^ Studies using detailed MRI-based stroke phenotyping have suggested the association may be confined to, or strongest in, patients with the lacunar stroke subtype.^10^

Based on the above data, we hypothesised that the *MTHFR* C677T variant may be a specific risk factor for SVD but not for other stroke subtypes. Lacunar infarcts are small and frequently not seen on computed tomography, and therefore, MRI is important for accurate diagnosis.^16^ Therefore, we determined whether the *MTHFR* polymorphism is associated with MRI-confirmed lacunar stroke. We compared these results with similar analyses from patients with cardioembolic and large artery stroke. In addition, we determined whether the same polymorphism was associated with MRI white matter hyperintensities, another marker of SVD. Because of the known association of hypertension with both tHcy and stroke, and in view of the positive results from the recent CSPPT study, we also stratified the analyses by hypertension status.

## Methods

### Stroke Populations

We included 1359 MRI-defined lacunar stroke cases from the UK young lacunar stroke DNA study, the Leuven Stroke Study (LSS), and the MRI-confirmed lacunar stroke collaboration (MCLSC), including cohorts from the UK, Germany, Italy, and Australia (Table I in the online-only Data Supplement). Lacunar stroke was defined as a clinical lacunar syndrome^17^ with an anatomically corresponding lacunar infarct on MRI (subcortical infarct ≤15 mm in diameter). All MRI scans were centrally reviewed by one physician (H.S. Markus). Exclusion criteria were stenosis >50% in the extra- or intracranial cerebral vessels; cardioembolic source of stroke, defined according to the Trial of Organization 10172 in Acute Stroke Treatment (TOAST) criteria^18^ as high or moderate probability; subcortical infarct >15 mm in diameter, as these can be caused by embolic mechanisms (striatocapsular infarcts); any other specific cause of stroke (eg, lupus anticoagulant, cerebral vasculitis, dissection, monogenic forms of stroke, eg, cerebral autosomal-dominant arteriopathy with subcortical infarcts and leukoencephalopathy [CADASIL]). Large-artery and cardioembolic stroke cases were obtained from Genetic Risk Factors for Leukoaraiosis Study (GENESIS), LSS, MCLSC, and the Wellcome Trust Case Control Immunochip Consortium (WTCCC2-Immunochip), including cohorts from the UK, Germany, Belgium, Italy, Sweden, Poland, Austria, and Australia. Cases were classified into stroke subtypes according to the pathophysiological TOAST classification,^18^ using clinical assessment, as well as brain and vascular imaging where available. Hypertension was defined as prescription of antihypertensives before stroke or systolic blood pressure >140 mm Hg or diastolic blood pressure >90 mm Hg >1 week post stroke. Fourteen thousand four hundred and forty-eight ancestry-matched controls were obtained from the same geographical location as the cases in each group. A description and characteristics of all cohorts are given in Table I in the online-only Data Supplement.

### White Matter Hyperintensity Volumes Population

The white matter hyperintensity (WMH) volume population (n=3670) was derived from the International Stroke Genetics Consortium (ISGC) WMH collaboration. This collaboration measured WMH volumes in patients with ischemic stroke from the UK, Italy, Belgium, Germany, Australia, and USA (Table II in the online-only Data Supplement). Inclusion criteria were >18 years of age, self-reported European ancestry, and a diagnosis of ischemic stroke. Exclusion criteria were any other cause of white matter disease, including CADASIL, vasculitis, and demyelinating and mitochondrial disorders. MRI scans were acquired as part of routine clinical practice for evaluation of ischemic stroke. Fluid-attenuated inversion recovery sequences were primarily used for leukoaraiosis analysis; however, in their absence, T2 sequences were used. All scans were quantitatively graded to obtain a WMH volume, which was normalized for intracranial volume. WMH volume was measured in the hemisphere contralateral to the infarct and doubled to obtain whole brain volumes. Patients with bilateral nonlacunar infarcts were excluded. All neuroimaging analyses have been previously described.^19^

### Genotyping

Direct genotyping of rs1801133 was performed in all cohorts except WTCCC2-Immunochip using commercially available arrays from Affymetrix or Illumina. In WTCCC2-Immunochip, rs1801133 was imputed from the 1000 Genomes integrated variant set (March 2012) using IMPUTE v2.^20^ The SNP was imputed with high accuracy (imputation info score =0.91). The SNP passed genotyping frequency thresholds (>97%) and was in Hardy–Weinberg equilibrium in all groups. To control for population stratification, individuals were removed that did not segregate with Hapmap II European populations based on ancestry informative principal component analysis using EIGENSTRAT software or multidimensional scaling in PLINK software, and ancestry-informative covariates were included in all analyses.^21,22^

### Statistical Analysis

In each cohort, logistic regression was performed to test for association of *MTHFR* C677T with MRI-defined lacunar stroke, cardioembolic stroke, and large artery stroke, assuming an additive model and adjusting for ancestry-informative principal components.

For each cohort, the association between WMH volume and *MTHFR* C677T was determined by performing linear regression of WMH volume on genotype dosages. Results across all cohorts were combined using a fixed-effects inverse variance weighted meta-analysis method. Subsequently, we repeated the analyses for MRI-defined lacunar stroke and WMH volume stratified by hypertension status.

We set our *P* value threshold for the main analyses to *P*<0.05 and then used a Bonferroni-corrected value (*P*=0.005) to assess significance in secondary analyses.

## Results

### Stroke Analyses

**Figure 1.**
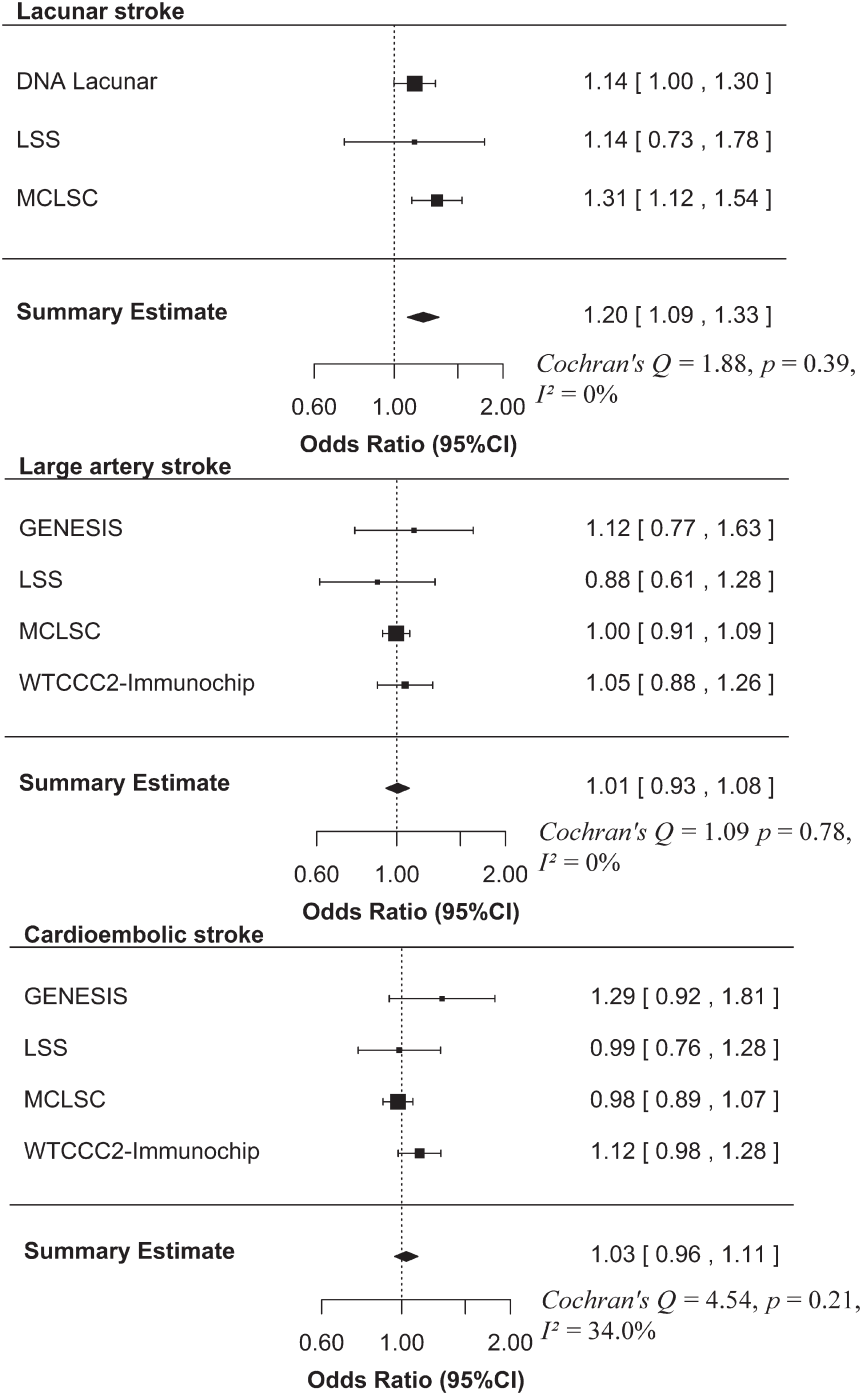
Forest plot for the association of *MTHFR* C677T with stroke subtypes. The size of the box is inversely proportional to the estimate variance of the effect estimator. GENESIS indicates Genetic Risk Factors for Leukoaraiosis Study; LSS, Leuven Stroke Study; MCLSC, magnetic resonance imaging–confirmed lacunar stroke collaboration; and WTCCC2-Immunochip, Wellcome Trust Case Control Immunochip Consortium.

*MTHFR* C677T was significantly associated with lacunar stroke (odds ratio [OR] 1.20, 95% CI 1.09–1.33; *P*=0.0003), but not with large-artery stroke (OR 1.01, 95% CI 0.93–1.08; *P*=0.88) or cardioembolic stroke (OR 1.03, 95% CI 0.96–1.11; *P*=0.44; Figure 1).

In the lacunar stroke cases, the association was most pronounced in homozygotes (OR 1.48, 95% CI 1.20–1.84 for TT versus CC; *P*=0.0003 and OR 1.17, 95% CI 1.01–1.36 for CT versus CC; *P*=0.03).

The overall prevalence of hypertension in the lacunar stroke cases was 72.6%. There were no differences in the prevalence of hypertension according to *MTHFR* genotype (72.0% in CC, 73.4% in CT, and 71.2% in TT). Stratifying the lacunar stroke cases for hypertension status demonstrated the association of *MTHFR* C677T with lacunar stroke was present in hypertensives (OR 1.24, 95% CI 1.11–1.38; *P*=0.0002), but not in normotensives (OR 1.09, 95% CI 0.92–1.29; *P*=0.30). In hypertensive and normotensive cases separately, the association of *MTHFR* C677T with lacunar stroke was again most pronounced in homozygotes (Table).

**Table.**
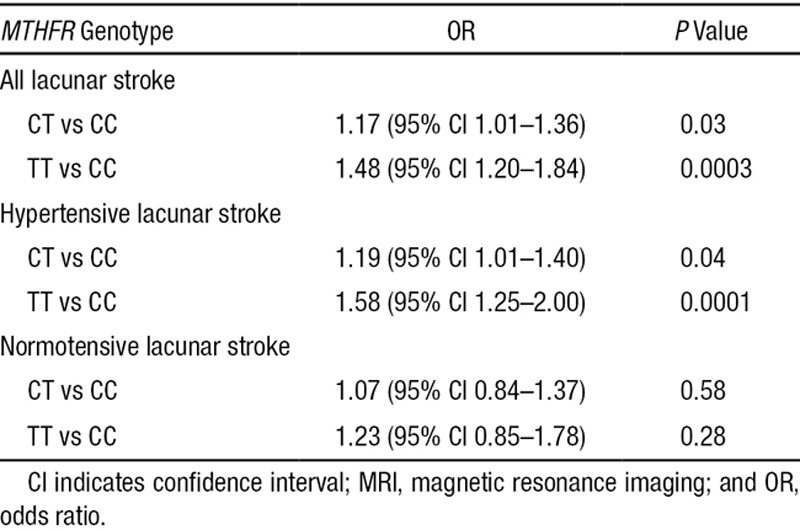
Association Between MTHFR C677T Genotypes and MRI-Defined Lacunar Stroke

### WMH Volumes Analyses

**Figure 2.**
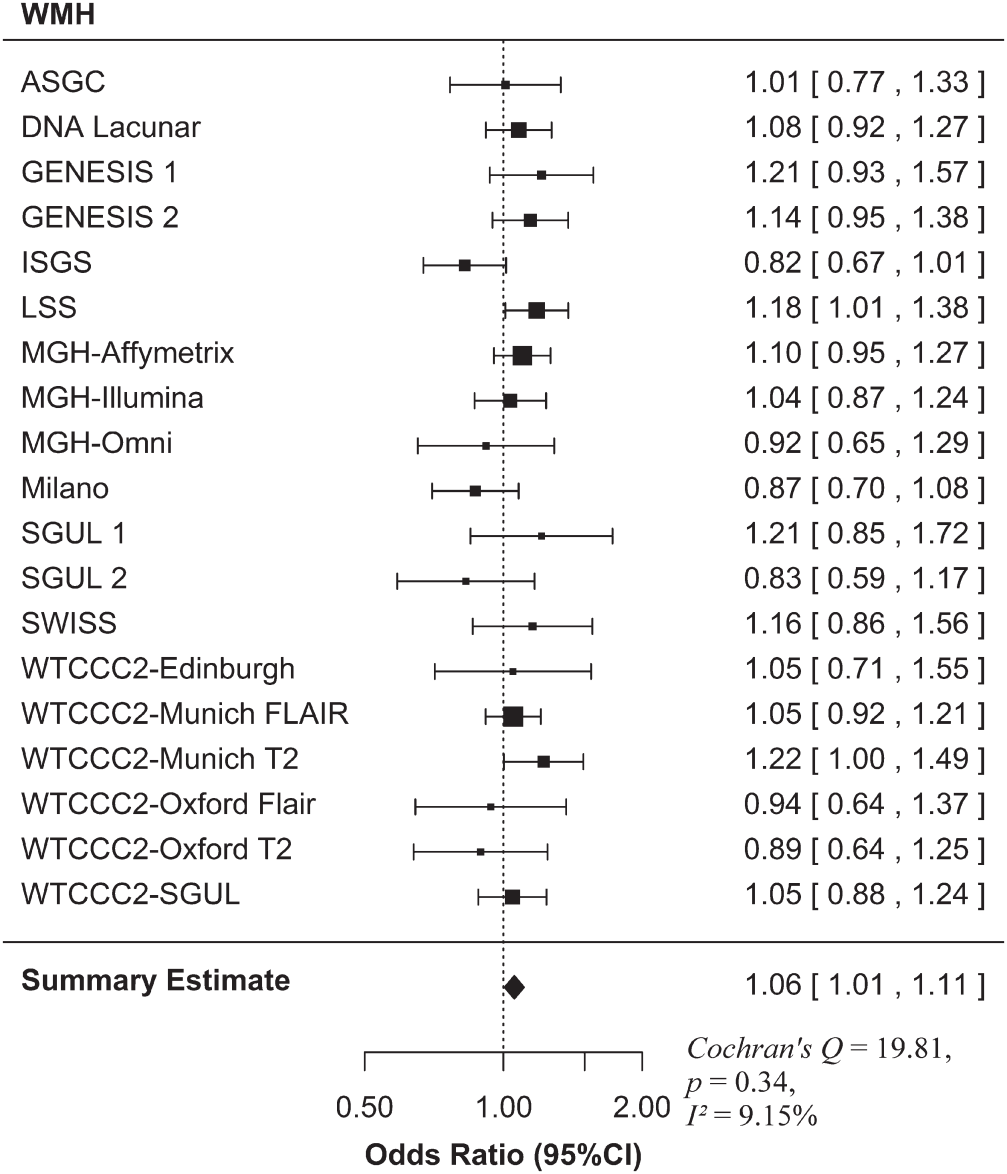
Forest plot for the association of *MTHFR* C677T with WMH. The size of the box is inversely proportional to the estimate variance of the effect estimator. ASGC indicates Australian Stroke Genetics Collaborative; GENESIS, Genetic Risk Factors for Leukoaraiosis Study; ISGS, Ischemic Stroke Genetics Study; LSS, Leuven Stroke Study; MGH, Massachusetts General Hospital; SGUL, St Georges University of London; SWISS, Sibling with Ischemic Stroke Study; and WTCCC2, Wellcome Trust Case-Control Consortium 2.

*MTHFR* C677T was significantly associated with WMH volume (OR 1.06, 95% CI 1.01–1.11; *P*=0.04; Figure 2). In the secondary analyses in which we stratified by hypertension status, there was a borderline association for either hypertensive cases (OR 1.06, 95% CI 1.00–1.13; *P*=0.05), although this did not pass the Bonferroni-corrected threshold, whereas there was no association in normotensive cases (OR 1.02, 95% CI 0.95–1.11; *P*=0.57).

## Discussion

In the present study, we showed that the tHcy-associated genetic variant *MTHFR* C677T was associated with lacunar stroke risk and cerebral SVD, but not for large artery or cardioembolic stroke, and that this association was restricted to patients with hypertension. Thereby, this study supports the hypothesis that homocysteine is a risk factor for specifically SVD and not for the other stroke subtypes.

Previous genetic association studies linking homocysteine to ischemic stroke have produced conflicting results.^15^ Such candidate studies may be influenced by publication bias, which is reduced in large multicentre GWAS studies. A recent analysis of 18 SNPs associated with tHcy reported equivocal results in 12 389 ischemic stroke cases (METASTROKE).^23^ There was no association between any SNPs associated with tHcy and all ischemic stroke or large artery or cardioembolic subtypes, consistent with this study. However, one SNP was associated with lacunar stroke (rs9369898, *MUT*), but no association was found with the MTHFR polymorphism.

Our study is the first large-scale study to include MRI-based phenotyping of lacunar stroke. The majority of stroke cases in previous studies have relied on CT brain imaging in combination of a diagnosis of a lacunar stroke syndrome. In ≤50% of cases, a clinical lacunar syndrome is caused by pathologies other than SVD.^24^

Another possible reason for the conflicting results between different studies is dietary folic acid fortification in certain populations. This was present in some cohorts in METASTROKE and might have attenuated any association with *MTHFR* C677T in METASTROKE.^15^ In the present study, folic acid fortification was not used in any of the included stroke cohorts, but was used in some of the included WMH volume cohorts. The association shown between *MTHFR* C677T and WMH volume in the present study might have been attenuated by the inclusion of these folic acid–fortified cohorts.

The SVD-specific effect of *MTHFR* C677T in the present study might reflect the effects of increased tHcy in SVD patients with this genetic variant. Although we did not assess tHcy in the present study and, therefore, cannot draw conclusions on this association in the present study, previous studies support this hypothesis of possible SVD-specific effect of increased tHcy. Secondary analyses of the VITATOPS trial suggested that homocysteine-lowering therapy may be associated with improved outcome in SVD (both lacunar stroke and WMH) but not in other stroke subtypes.^3^ In the VITATOPS trial, in which lacunar stroke subtyping was largely based on a clinical stroke syndrome with computed tomography imaging, a borderline significant reduction in recurrent stroke occurred in patients with SVD; based on our results, one could hypothesise that this treatment effect might be stronger in MRI-confirmed lacunar stroke. Consistent with this specific effect in SVD are the results of an MRI substudy in 359 individuals from VITATOPS; although no association was found in the group as a whole, in a subanalysis of patients with MRI evidence of severe SVD at baseline, B vitamin supplementation was associated with a significant reduction in WMH volume change.^11^ Two meta-analyses on previous genetic association studies linking MTHFR with WMH on MRI could not confirm an association between MTHFR and WMH.^25,26^ Individual studies that were included in these meta-analyses had only small number of patients, and it was suggested that much larger studies would be needed to detect an association.^25^ In the present study, we included twice as many subjects as the largest study in the previous meta-analyses that assessed WMH on a dichotomous scale and three times as many subjects as the study that assessed WMH volume.

We found that the association of *MTHFR* C677T with lacunar stroke was restricted to hypertensive individuals. One possible explanation for this finding exclusively in hypertensive individuals might be that the association acts through increased susceptibility to, or interaction with, high blood pressure. Interestingly, the recent large primary prevention CSPPT trial showed a benefit of homocysteine-lowering therapy in reducing stroke risk in hypertensive individuals from a population in which folic acid fortification was not occurring.^7^

The major strength of our study is the confirmation of all lacunar strokes by MRI and the relatively large sample size. Furthermore, the approach of using a genetic proxy for tHcy reduces the likeliness of reversed causality and residual confounding compared with previous observational studies. The relationship between tHcy and stroke in observational studies might be confounded by unmeasured or not adequately measured factors (eg, other dietary factors) that are causally associated with stroke. In contrast, genetic studies rely on the fact that genetic variants are fixed from conception and are not influenced by other traits.

Moreover, the stroke cohorts were derived from countries in which folic acid fortification was not implemented at the time of stroke, which maximized the chances of demonstrating an effect for *MTHFR* C677T. We also included an analysis of WMH volume in ischemic stroke patients. Stroke patients represent an enriched population in whom WMH are increased. It has been shown, however, that the genetic factors underlying WMH in ischemic stroke patients seem to be similar to those in population-based studies of WMH.^19^

A potential limitation of the present study is that we did not have independent replication cohorts available to validate our findings, and therefore, future studies are warranted to confirm these interesting findings. Furthermore, although *MTHFR* C677T is strongly associated with tHcy in other studies,^12,14^ we could not also directly assess the association of tHcy with stroke subtype in the present study because tHcy measurements were not available in all of our cohorts.

In summary, we showed that *MTHFR* C677T was associated with lacunar stroke in hypertensive individuals, supporting a possible causal role for homocysteine in the pathogenesis of cerebral SVD. Our results suggest that any future trials investigating the benefit of lowering homocysteine in stroke patients should focus on the SVD subtype and that they should incorporate MRI-based diagnosis.

## Acknowledgments

We thank all study staff and participants for their important contributions. Study-specific collaborators are reported in the online-only Data Supplement.

## Sources of Funding

Collection of the UK Young Lacunar Stroke DNA Study (DNA Lacunar) was primarily supported by the Wellcome Trust (WT072952) with additional support from the Stroke Association (TSA 2010/01). Genotyping of the DNA Lacunar samples and Dr Traylor were supported by a Stroke Association Grant (TSA 2013/01). Genotyping of WTCCC2 ischemic stroke study was funded by the Wellcome Trust. The Oxford Vascular Study has been funded by Wellcome Trust, Wolfson Foundation, UK Stroke Association, British Heart Foundation, Dunhill Medical Trust, National Institute of Health Research (NIHR), Medical Research Council, and the NIHR Oxford Biomedical Research Centre. Funding for the genotyping at Massachusetts General Hospital was provided by the Massachusetts General Hospital-Deane Institute for the Integrative Study of Atrial Fibrillation and Stroke and the National Institute of Neurological Disorders and Stroke (U01 NS069208). Dr Rutten-Jacobs was supported by a Stroke Association/British Heart Foundation programme grant (TSA BHF 2010/01). Dr Adib-Samii was supported by a Medical Research Council (United Kingdom) training fellowship. Drs Markus and Bevan are supported by the National Institute for Health Research Cambridge University Hospitals Comprehensive Biomedical Research Centre. Dr Markus is supported by a National Institute for Health Research Senior Investigator award. Dr Thijs is supported by a Clinical Investigator Grant from the scientific research fund, Fonds Wetenschappelijk Onderzoek Flanders. Dr Levi is supported by a National Health and Medical Research Council (NHMRC Australia) Practitioner Fellowship and the Australian Stroke Genetics Collaboration has received Project Grant support from the NHMRC (App 1010287). Dr Rost was supported by a National Institute of Neurological Disorders and Stroke grant (R01 NS082285-01). Professor Rothwell is in receipt of an NIHR Senior Investigator Award and a Wellcome Trust Senior Investigator Award. We also acknowledge the use of the facilities of the Acute Vascular Imaging Centre, Oxford, and the Cardiovascular Clinical Research Facility, Oxford. The sponsors of the study had no role in the study design, data collection, data analysis, interpretation, writing of the article, or the decision to submit the article for publication.

## Disclosures

None.

## Supplementary Material
