# Supplementary material for: Association of MTHFR C677T Genotype With Ischemic Stroke Is Confined to Cerebral Small Vessel Disease Subtype
Source: Stroke. 2016 Feb 22;47(3):646–51. doi: 10.1161/STROKEAHA.115.011545 (PMC4760380; doi:10.1161/STROKEAHA.115.011545)
Supplement: Supplementary file 1 [file str-47-646-s001.pdf]

SUPPLEMENTAL MATERIAL

Association of *MTHFR* C677T genotype with ischemic stroke is confined to cerebral small vessel disease subtype

Loes CA Rutten-Jacobs, PhD[1], Matthew Traylor, PhD[1,2], Poneh Adib-Samii, MBBS[3], Vincent Thijs, MD[4], Cathie Sudlow, FRCP[5], Peter M Rothwell, FMedSci[6], Giorgio Boncoraglio, MD[7], Martin Dichgans, MD[8], James Meschia, MD[9], Jane Maguire, PhD[10], Christopher Levi, MD[10], Natalia S Rost, MD[11], Jonathan Rosand, MD[11], Ahamad Hassan, FRCP[12], Steve Bevan, PhD[13], Hugh S Markus, FRCP[1]

Affiliations
[1] University of Cambridge, Department of Clinical Neurosciences, Cambridge, UK
[2] Department of Medical & Molecular Genetics, King's College London, London, UK
[3] Stroke and Dementia Research Centre, St George's University of London, London, UK
[4] KULeuven Department of Experimental Neurology and Leuven Research Institute for Neuroscience and Disease, University of Leuven, and Laboratory of Neurobiology, Vesalius Research Center, VIB, Leuven, Belgium and Department of Neurology, Austin Health and Florey Institute of Neuroscience and Mental Health, Heidelberg, Australia
[5] Division of Clinical Neurosciences, Neuroimaging Sciences and Institute of Genetics and Molecular Medicine, University of Edinburgh, Edinburgh, UK
[6] Stroke Prevention Research Unit, Nuffield Department of Neuroscience, University of Oxford, UK
[7] Department of Cerebrovascular Diseases, Fondazione IRCCS Istituto Neurologico "Carlo Besta", Milano, Italy
[8] Institute for Stroke and Dementia Research, Klinikum der Universität München, Ludwig-Maximilians-University Munich and Munich Cluster of Systems Neurology, SyNergy, Munich, Germany
[9] Department of Neurology, Mayo Clinic, Jacksonville, USA
[10] Centre for Clinical Epidemiology and Biostatistics, Hunter Medical Research Institute and School of Medicine and Public Health, University of Newcastle, NSW, Australia
[11] Center for Human Genetic Research and Department of Neurology, Massachusetts General Hospital, Boston, USA
[12] Department of Neurology, Leeds General Infirmary, Leeds Teaching Hospitals NHS Trust, Leeds, UK
[13] School of Life Science, University of Lincoln, UK

Corresponding Author:
Loes Rutten-Jacobs
Department of Clinical Neurosciences
University of Cambridge
Cambridge
CB2 0QQ
LR406@medschl.cam.ac.uk

Cohorts description

UK Young Lacunar Stroke DNA Study (DNA Lacunar)

DNA Lacunar is a multicentre cohort study, which constitutes a large DNA resource of young patients with well phenotyped lacunar stroke and stroke-free community controls. Between 2005 and 2012, 1030 white patients of European ancestry with lacunar stroke, aged ≤ 70 years, were recruited from 72 specialist stroke centres throughout the UK. All patients underwent brain MRI, imaging of the carotid arteries and ECG. Echocardiography was performed when appropriate. All MRI's and clinical histories were reviewed centrally by one experienced stroke physician.
970 Unrelated Caucasian controls, free of clinical cerebrovascular disease, were obtained by random sampling from general practice lists from the same geographical location as the patients. Sampling was stratified for age and sex.

MRI-confirmed ischemic stroke collaboration (MCISC)

Wellcome Trust Case-Control Consortium 2 (WTCCC2)
The WTCCC2 samples were genotyped as part of the WTCCC 2 ischemic stroke study. Stroke cases were recruited from three centres in the UK (St. George's University London, Oxford and Edinburgh) and one centre in Germany, University and Klinikum Großhadern, Ludwig-Maximilians-University, Munich
WTCCC2-UK: The St George's Stroke Study consecutively recruited ischemic stroke patients attending cerebrovascular services in London between 1995 and 2008. All patients had clinically relevant diagnostic workup performed, including brain imaging with computed tomography (CT) and/or magnetic resonance imaging (MRI) as well as ancillary diagnostic investigations including duplex ultrasonography of the carotid and vertebral arteries, echocardiography,
Holter monitoring, magnetic resonance angiography (MRA), CT-angiography
(CTA) and blood tests. The Oxford Vascular Study recruited patients with acute ischemic stroke or transient ischemic attack (TIA) with evidence of infarction on brain imaging between 2002 and 2008 as part of a population-based stud. All cases were phenotyped by one experienced stroke neurologist with review of original imaging. The Edinburgh Stroke Study prospectively recruited consecutive stroke inpatients and outpatients between 2002 and 2005. An experienced stroke physician assessed each patient as soon as possible after the stroke, prospectively recording demographic and clinical details, including vascular risk factors and results of brain imaging and other investigations
WTCCC2-Germany: The Munich study recruited consecutively between 2002 and 2008, from a single Stroke Unit with a high rate of MR imaging (>80%) (n=1383). All subjects were over 18 years of age, of self-reported European ancestry and with a diagnosis of ischemic stroke classified according to TOAST by an experienced neurologist or stroke physician. All patients had brain imaging as well as ancillary diagnostic investigations where clinically relevant.
Controls for the UK samples were drawn from shared WTCCC controls obtained from the 1958 Birth Cohort. This is a prospectively collected cohort of individuals born in 1958 (http://www.b58cgene.sgul.ac.uk/), and ascertained as part of the national child development study (http://www.cls.ioe.ac.uk/studies.asp?section=000100020003). Data from this cohort are available as a common control set for a number of genetic and epidemiological studies. For the German samples controls were Caucasians of German origin participating into the population KORAgen study (www.gsf.de/kora/en/english.html). This survey represents a gender- and age stratified random sample of all German residents of the Augsburg area and

consists of individuals 25 to 74 years of age, with about 300 subjects for each 10-year increment. All controls were free of a history of stroke or transient ischemic attack.

Besta Stroke Study (Milano)
This study includes consecutive Italian patients referred to Besta Institute from 2000 to 2009 with stroke and included in the Besta Cerebrovascular Diseases Registry (CEDIR). Ischemic stroke cases, first ever or recurrent, confirmed on brain imaging, were selected for this study. An experienced stroke neurologist assessed all cases.

Australian Stroke Genetics Collaborative (ASGC)
Stroke cases comprised European-ancestry patients admitted to four clinical centres across Australia (The Neurosciences Department at Gosford Hospital, Gosford, New South Wales (NSW); the Neurology Department at John Hunter Hospital, Newcastle, NSW; The Queen Elizabeth Hospital, Adelaide; and the Royal Perth Hospital, Perth) between 2003 and 2008. Stroke was defined by WHO criteria as a sudden focal neurologic deficit of vascular origin, lasting more than 24 hours and confirmed by brain imaging. Other investigative tests such as electrocardiogram, carotid Doppler and trans-oesophageal echocardiogram were conducted to define stroke aetiology as clinically appropriate.

Leuven Stroke Study

Patients with cerebral ischemia, defined as a clinical stroke with imaging confirmation or a TIA with a new ischemic lesion on diffusion weighted MRI, who were admitted to the Stroke Unit of the University Hospitals in Leuven were enrolled. All patients underwent brain imaging and a standardized protocol including carotid ultrasound or CT angiography and cardiac examination (echocardiography and Holter monitoring) in all patients.
Control individuals were selected from the same population and were either spouses of patients with multiple sclerosis, amyotrophic lateral sclerosis or stroke or healthy community dwelling subjects partially from the Leuven University Gerontology Database. Controls either confirmed they never had a stroke or TIA or responded negative to any item of the Verification of Stroke Free Status questionnaire.

GENESIS

This study recruited patients attending cerebrovascular services at St. George’s Hospital, London between 2011-2013. All patients had clinically relevant diagnostic workup performed, including brain imaging with magnetic resonance imaging (MRI) as well as ancillary diagnostic investigations including duplex ultrasonography of the carotid and vertebral arteries, echocardiography,
Holter monitoring, magnetic resonance angiography (MRA), CT-angiography (CTA) and blood tests

WTCCC2-Immunochip

Bio-Repository of DNA in Stroke (BRAINS): London
The Bio-Repository of DNA in Stroke (BRAINS) is an international study recruiting highly phenotyped patients with stroke. For the purposes of the current work all patients were Caucasians. Diagnosis of stroke was confirmed using positive imaging (MRI or CT) and ischemic stroke subtypes were assigned using TOAST criteria, based on clinical, imaging and risk factor data. The cohort has been described in detail elsewhere (Yadav S, Schanz R,

Maheshwari A, et al. Bio-Repository of DNA in stroke (BRAINS): a study protocol. BMC Med Genet 2011; 12: 34).

Glasgow: Scotland
Patients with ischemic stroke attending the cerebrovascular service of the Western Infirmary, Glasgow, were recruited between 1990 and 2004 as part of an ongoing study of genetic and circulating biomarkers in stroke. All patients underwent brain imaging and extracranial carotid ultrasound in accordance with a standard clinical protocol. The study was approved by the West Ethics Committee.

Controls for the UK samples were drawn from shared WTCCC controls obtained from the 1958 Birth Cohort. This is a prospectively collected cohort of individuals born in 1958 (http://www.b58cgene.sgul.ac.uk/), and ascertained as part of the national child development study (http://www.cls.ioe.ac.uk/studies.asp?section=000100020003). Data from this cohort are available as a common control set for a number of genetic and epidemiological studies.

Lund Stroke Register, Sweden
Lund Stroke Register (LSR) since 2001 continuously enrolls patients aged 18 and older with first-ever stroke, living in the primary uptake area of Skane University Hospital, Lund. The study is mainly hospital-based but has a good coverage of the whole geographical population.28 All included patients are examined with CT/MR or autopsy of the brain. When clinically indicated, the patients are examined with ultrasound imaging of carotid arteries, echocardiography, and angiography. In this study, first-ever ischemic stroke patients from LSR between 2001 and 2004 were included. All patients were assessed by a neurologically trained physician regarding stroke type. Informed consent was obtained from all individuals or when they were not able to respond from their next-of-kin. The study was approved by the Ethics Committee of Lund University. Biobank services were performed at Region Skane Competence Centre (RSKC Malmo), Malmo University Hospital, Malmo, Sweden.

Swedish Control Samples
Controls for the Lund cases were provided by the Swedish SLE network. Controls were healthy blood donors from the geographical areas of Uppsala, Stockholm and Lund. The studies were approved by the regional ethics boards and all subjects gave their informed consent to participate. Genotyping of the Swedish control samples was performed at the SNP&SEQ technology platform in Uppsala, Sweden (www.genotyping.se).

Munich: Germany
Cases were consecutive European Caucasians recruited from a single dedicated Stroke Unit at the Department of Neurology, Klinikum Groβhadern, Ludwig-Maximilians-University, Munich. Ischemic stroke subtypes were determined according to TOAST criteria based on relevant clinical and imaging data.

German control samples
German healthy control individuals were obtained from the PopGen biobank [Krawczak et al., Community Genet 9:55-61, 2006]. Written, informed consent was obtained from all study participants and all protocols were approved by the institutional ethical review committee of the participating centre. The panel is a cross-sectional control cohort from the Kiel area in Northern Germany. More than 300 phenotypes were collected for the cohort and a 3-year follow has recently been completed. All data and biomaterials are accessible via the PopGen biobank. The Genotyping was part of the German National Genome Research Network

(NGFN) GWAS initiative and performed by the Institute of Clinical Molecular Biology (Christian-Albrechts-University of Kiel).

Poland:Krakow
Patients were recruited in the stroke unit of the Jagiellonian University in Krakow, Poland (a single-center study). All stroke patients and controls were >18 years of age and were white. All patients had clinically relevant diagnostic workup performed, including brain imaging with computed tomography (CT) (100%) and/or magnetic resonance imaging (MRI) (8%) as well as ancillary diagnostic investigations including duplex ultrasonography of the carotid and vertebral arteries (85.2%), echocardiography (54.8%). MR-angiography, CT-angiography Holter monitoring, transesophageal echocardiography and blood tests for hypercoagulability were performed were indicated. Patients were classified into etiologic subtypes according to the Trial of Org 10172 in Acute Stroke Treatment (TOAST).
The control group included unrelated subjects taken from the population of southern Poland. Control subjects had no apparent neurological disease based on the findings in a structured questionnaire and a neurological examination. The study was approved by local research ethics committees and informed consent was obtained from all participants.

White Matter Hypertensities collaboration International Stroke Genetics Consortium

St Georges University of London (SGUL)
This study recruited patients attending cerebrovascular services at St. George's Hospital, London between 2007-2011. All patients had clinically relevant diagnostic workup performed, including brain imaging with magnetic resonance imaging (MRI) as well as ancillary diagnostic investigations including duplex ultrasonography of the carotid and vertebral arteries, echocardiography,
Holter monitoring, magnetic resonance angiography (MRA), CT-angiography
(CTA) and blood tests

Massachusetts General Hospital (MGH)
Cases presenting with ischemic stroke and admitted to the Massachusetts General Hospital (MGH) Stroke Unit through the Emergency Department, or evaluated in the MGH Neurology outpatient clinics, as well as on the inpatient Medical and Vascular Surgical services from January 2003 to July 2008. Ischemic stroke was defined as either (1) a radiographically proven (head CT or MRI) infarct associated with the appropriate clinical stroke syndrome, or (2) a fixed neurological deficit persisting more than 24 hours, consistent with a vascular pattern of involvement and without radiographic evidence of demyelinating or other non-vascular disease. All subjects were evaluated by a neurologist upon presentation and clinical and laboratory data were collected during the admission for qualifying ischemic stroke event. All patients had acute brain imaging as well as ancillary diagnostic investigations: cervical and intracranial vessel imaging using CT or MR angiography (75%), cervical ultrasound (24%), echocardiography (86%), and Holter monitoring (16%).

Ischemic Stroke Genetics Study (ISGS)
Ischemic Stroke Genetics Study (ISGS) was a 5-center, prospective, case-control study of first-ever ischemic stroke cases in the United States. All affected individuals had WHO-defined stroke confirmed by a study neurologist to be ischemic on the basis of head CT or brain MRI. Peripheral blood DNA samples were collected between May 2003 and September 2008.

Sibling with Ischaemic Stroke Study (SWISS)
This is a prospective, multicentre study of sibling pairs with first-ever or recurrent ischemic stroke. Probands were recruited from 70 clinical centres across the US and Canada. Ischemic stroke affected and unaffected siblings were recruited primarily using proband-initiated contact. All affected individuals had WHO-defined stroke confirmed by a study neurologist to be ischemic on the basis of brain imaging. Peripheral blood DNA samples were collected between October 2000 and December 2009.

Appendix

UK Young Lacunar Stroke DNA Study collaborators

Study managers: Josie Monaghan; Alan Zanich, Samantha Febrey, Eithne Smith, Jenny Lennon, St George's University of London

Participating centres (number of enrolled patients per centre; local investigators): Aberdeen Royal Infirmary, Aberdeen (12; Mary Macleod). Addenbrooke's Hospital, Cambridge (54; Jean-Claude Baron, Elizabeth Warburton, Diana J Day, Julie White). Airedale General Hospital, Steeton (4; Samantha Mawer). Barnsley Hospital, Barnsley (3; Mohammad Albazzaz, Pravin Torane, Keith Elliott, Kay Hawley). Bart's and the London, London (2; Patrick Gompertz). Basingstoke and North Hampshire Hospital, Basingstoke (13; Elio Giallombardo, Deborah Dellafera). Blackpool Victoria Hospital, Blackpool (11; Mark O'Donnell). Bradford Royal Infirmary, Bradford (1; Chris Patterson). Bristol Royal Infirmary, Bristol (8; Sarah Caine). Charing Cross Hospital, London (12; Pankaj Sharma). Cheltenham General and Gloucester Royal Hospitals, Cheltenham and Gloucester (10; Dipankar Dutta). Chesterfield Royal Hospital, Chesterfield (4; Sunil Punnoose, Mahmud Sajid). Countess of Chester Hospital, Chester (22; Kausik Chatterjee). Derriford Hospital, Plymouth (4; Azlisham Mohd Nor). Dorset County Hospital NHS Foundation Trust, Dorchester (6; Rob Williams). East Kent Hospitals University NHS Foundation Trust, Kent (22; Hardeep Baht, Guna Gunathilagan). Eastbourne District General Hospital, Eastbourne (4; Conrad Athulathmudali). Frenchay Hospital, Bristol (1; Neil Baldwin). Frimley Park Hospital NHS Foundation Trust, Frimley (6; Brian Clarke). Guy's and St Thomas' Hospital, London (14; Tony Rudd). Institute of Neurology, London (25; Martin Brown). James Paget University Hospital, Great Yarmouth (1; Peter Harrison). King's College Hospital, London (16; Lalit Kalra). Leeds Teaching Hospitals NHS Trust, London (125; Ahamad Hassan). Leicester General Hospital and Royal Infirmary, Leicester (9; Tom Robinson, Amit Mistri). Luton and Dunstable NHSFT University Hospital, Luton (16; Lakshmanan Sekaran, Sakthivel Sethuraman, Frances Justin). Maidstone andTunbridge Wells NHS Trust (3; Peter Maskell). Mayday University Hospital, Croydon (14; Enas Lawrence). Medway Maritime Hospital, Gillingham (5; Sam Sanmuganathan). Milton Keynes Hospital, Milton Keynes (1; Yaw Duodu). Musgrove Park Hospital, Taunton (9; Malik Hussain). Newcastle Hospitals NHS Foundation Trust, Newcastle upon Tyne (12; Gary Ford). Ninewells Hospital, Dundee (5; Ronald MacWalter). North Devon District Hospital, Barnstaple (8; Mervyn Dent). Nottingham University Hospitals, Nottingham (17; Philip Bath, Fiona Hammonds). Perth Royal Infirmary, Perth (2; Stuart Johnston). Peterborough City Hospital, Peterborough (1; Peter Owusu-Agyei). Queen Elizabeth Hospital, Gateshead (5; Tim Cassidy, Maria Bokhari). Radcliffe Infirmary, Oxford (5; Peter Rothwell). Rochdale Infirmary, Rochdale (4; Robert Namushi). Rotherham General Hospital, Rotherham (1; James Okwera). Royal Cornwall Hospitals NHS Trust, Truro (11; Frances Harrington, Gillian Courtauld). Royal Devon and Exeter Hospital, Exeter (22; Martin James). Royal Hallamshire Hospital, Sheffield (1; Graham Venables). Royal Liverpool University Hospital and Broadgreen Hospital, Liverpool (9; Aravind Manoj). Royal Preston Hospital, Preston (18; Shuja Punekar). Royal Surrey County Hospital, Guildford (23; Adrian Blight, Kath Pasco). Royal Sussex County Hospital, Brighton (14; Chakravarthi Rajkumar, Joanna Breeds). Royal United Hospital, Bath (6; Louise Shaw, Barbara Madigan). Salford Royal Hospital, Salford (16; Jane Molloy). Southampton General Hospital, Southampton (1; Giles Durward). Southend Hospital, Westcliff-on-Sea (26; Paul Guyler). Southern General Hospital, Glasgow (34; Keith Muir,

Wilma Smith). St George’s Hospital, London (108; Hugh Markus). St Helier Hospital, Carshalton (10; Val Jones). Stepping Hill Hospital, Stockport (4; Shivakumar Krishnamoorthy). Sunderland Royal Hospital, Sunderland (1; Nikhil Majumdar). The Royal Bournemouth Hospital, Bournemouth (15; Damian Jenkinson). The Walton Centre, Liverpool (15; Richard White). Torbay Hospital, Torquay (19; Debs Kelly). University Hospital Aintree, Liverpool (19; Ramesh Durairaj). University Hospital of North Staffordshire, Stoke-on-trent (16; David Wilcock). Wansbeck General Hospital and North Tyneside Hospital, Ashington and North Shields (6; Christopher Price). West Cumberland Hospital, Whitehaven (6; Olu Orugun, Rachel Glover). West Hertfordshire Hospital, Watford (20; David Collas). Western General Hospital, Edinburgh (12; Cathie Sudlow). Western Infirmary, Glasgow (33; Kennedy R. Lees, Jesse Dawson). Wycombe Hospital and Stoke Mandeville, High Wycombe (20; Dennis Briley and Matthew Burn). Yeovil District Hospital, Yeovil (46; Khalid Rashed). York Teaching Hospital, York (1; John Coyle).

Table I Stroke cohorts

Cohort	N	Mean age (sd)	% Male	% Hypertensive
DNA Lacunar & GENESIS				
Lacunar stroke	998	57.3 (9.6)	704 (71%)	705 (71%)
Large artery stroke	64	70.1 (11.6)	44 (69%)	
Cardioembolic stroke	80	73.5 (14.6)	47 (59%)	
Controls	970	59.7 (4.3)	510 (53%)	NA
MCLSC				
Lacunar stroke	319	68.5 (11.8)	190 (60%)	262 (82%)
Large artery stroke	1338	68.9 (11.9)	904 (68%)	906 (68%)
Cardioembolic stroke	1094	73.7 (13.8)	558 (51%)	761 (70%)
Controls	7623	62.5 (10.2)	4002 (52%)	NA
LSS				
Lacunar stroke	42	65.5 (13.9)	29 (69%)	26 (62%)
Large artery stroke	70		43 (61%)	48 (69%)
Cardioembolic stroke	157		96 (61%)	98 (62%)
Controls	455	55.7 (14.5)	212 (47%)	NA
WTCCC2-Immunochip				
Large artery stroke	352	69.1 (12.2)	220 (62%)	234 (67%)
Cardioembolic stroke	639	75.7 (11.8)	339 (53%)	417 (67%)
Controls	5401	NA	2304 (43%)	NA

Abbreviations: DNA Lacunar, UK Young Lacunar Stroke DNA Study; GENESIS, Genetic Risk factors for Leukoaraiosis study; MCLSC, MRI-confirmed lacunar stroke collaboration; LSS, Leuven Stroke Study, WTCCC2-Immunochip, Wellcome Trust Case-Control Consortium II Immunochip

Table II WMH study populations

Centre	Country	N	Mean age (sd)	% Male	% Hypertensive
Milano	Italy	151	57 (14)	60%	57%
WTCCC2-Edinburgh	UK	64	68 (13)	50%	72%
WTCCC2-Munich FLAIR	Germany	447	66 (12)	66%	72%
WTCCC2-Munich T2	Germany	203	67 (12)	55%	67%
WTCCC2-Oxford Flair	UK	65	65 (15)	54%	65%
WTCCC2-Oxford T2	UK	75	67 (13)	59%	68%
WTCCC2-SGUL	UK	323	70 (14)	63%	77%
GENESIS 1	UK	121	67 (14)	67%	62%
GENESIS 2	UK	228	69 (15)	58%	76%
SGUL 1	UK	70	70 (13)	61%	61%
SGUL 2	UK	57	68 (14)	58%	72%
DNA Lacunar	UK	303	57 (9)	72%	68%
LSS	Belgium	361	66 (15)	58%	59%
MGH-Affymetrix	US	476	67 (14)	60%	64%
MGH-Omni	US	84	64 (15)	63%	68%
MGH-Illumina	US	228	66 (15)	64%	61%
ASGC	Australia	96	65 (13)	57%	77%
ISGS	US	207	68 (14)	62%	61%
SWISS	US	111	66 (11)	48%	74%
Overall		3670			

Abbreviations: Milano, Besta Stroke Register; WTCCC2, The Wellcome Trust Case-Control Consortium II; GENESIS, Genetic Risk factors for Leukoaraiosis study; SGUL, St Georges University of London; DNA Lacunar, UK Young Lacunar Stroke DNA Study; LSS, Leuven Stroke Study; MGH, Massachusetts General Hospital; ASGC, Australian Stroke Genetics Collaborative; ISGS, Ischemic Stroke Genetics Study; SWISS, Sibling with Ischaemic Stroke Study
